# A Novel Cipher-Based Data Encryption with Galois Field Theory

**DOI:** 10.3390/s23063287

**Published:** 2023-03-20

**Authors:** Mohammad Mazyad Hazzazi, Sasidhar Attuluri, Zaid Bassfar, Kireet Joshi

**Affiliations:** 1 Department of Mathematics, College of Science, King Khalid University, Abha 61413, Saudi Arabia; 2 Software Developer Applications, 671 E Royal Ln, APT 1074, Irving, TX 75039, USA; 3 Department of Information Technology, University of Tabuk, Tabuk 71491, Saudi Arabia; 4 Department of CSE, Graphic Era Deemed to be University, Dehradun 248002, India

**Keywords:** encryption, decryption, Black Widow Optimization Galois field, advanced encryption standard, discrete cosine transform, cryptography

## Abstract

Both the act of keeping information secret and the research on how to achieve it are included in the broad category of cryptography. When people refer to “information security,” they are referring to the study and use of methods that make data transfers harder to intercept. When we talk about “information security,” this is what we have in mind. Using private keys to encrypt and decode messages is a part of this procedure. Because of its vital role in modern information theory, computer security, and engineering, cryptography is now considered to be a branch of both mathematics and computer science. Because of its mathematical properties, the Galois field may be used to encrypt and decode information, making it relevant to the subject of cryptography. The ability to encrypt and decode information is one such use. In this case, the data may be encoded as a Galois vector, and the scrambling process could include the application of mathematical operations that involve an inverse. While this method is unsafe when used on its own, it forms the foundation for secure symmetric algorithms like AES and DES when combined with other bit shuffling methods. A two-by-two encryption matrix is used to protect the two data streams, each of which contains 25 bits of binary information which is included in the proposed work. Each cell in the matrix represents an irreducible polynomial of degree 6. Fine-tuning the values of the bits that make up each of the two 25-bit binary data streams using the Discrete Cosine Transform (DCT) with the Advanced Encryption Standard (AES) Method yields two polynomials of degree 6. Optimization is carried out using the Black Widow Optimization technique is used to tune the key generation in the cryptographic processing. By doing so, we can produce two polynomials of the same degree, which was our original aim. Users may also use cryptography to look for signs of tampering, such as whether a hacker obtained unauthorized access to a patient’s medical records and made any changes to them. Cryptography also allows people to look for signs of tampering with data. Indeed, this is another use of cryptography. It also has the added value of allowing users to check for indications of data manipulation. Users may also positively identify faraway people and objects, which is especially useful for verifying a document’s authenticity since it lessens the possibility that it was fabricated. The proposed work achieves higher accuracy of 97.24%, higher throughput of 93.47%, and a minimum decryption time of 0.0047 s.

## 1. Introduction

Learning how to encrypt data using cyphers and then decrypt it with a secret key was the focus of the field of study known as cryptography [1]. It includes all methods of encrypting data such that unauthorized parties can’t read it or reverse it using computational means. The most basic role that cryptography may play is in the secure transfer of data between parties, whereby only the intended recipients have access to the sent data.

In this part, we’ll focus on a specific kind of cryptography that relies on numerical representations of data and the arithmetical manipulation of those representations. Additional services, such as [2]

**Figure 1:**
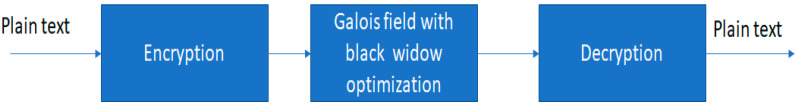
Cryptographic Processing.

- Making sure the message hasn’t been tampered with and came from a legitimate source, often known as verifying its integrity.
- To authenticate someone or something is to verify their identity or authenticity. But, let’s discuss the common applications of cryptography. The “plaintext” or “clear text” of a message is what it says when taken in its “actual form.” Cypher text is a term that refers to information that has been scrambled. Encryption is the method used to transform plaintext into unreadable code. Figure 1 depicts this. Some people even go so far as to call decryption the “opposite” of encryption [3].

According to [4] Cryptanalysts are the people who try to crack the codes that cryptographers have constructed. These two activities are always competing with one another. Each and every cryptographic system necessitates a secret value and a corresponding algorithm (key). It is quite tough to maintain developing innovative algorithms that will allow for the reversible scrambling of information, and it is difficult to swiftly explain a newly developed algorithm to the person with whom someone would want to begin interacting securely. Scrambled data cannot be deciphered without a key that is also part of the data itself.

In a well-designed cryptosystem, knowing the algorithm is absolutely OK for anybody, even the bad guys (cryptanalysts), since it is worthless without also possessing the key. Due to the fact that understanding the algorithm is meaningless without also possessing the key [5]. A key is used because it facilitates the clustering that is necessary for a combination lock. Although the principle of a combination lock is familiar (to open the lock, one must dial in a series of secret numbers in the proper order), a combination lock cannot be opened by brute force without knowledge of the combination.

### Secret Key Cryptography

The use of a secret key to encrypt data is also known as symmetric cryptography or traditional cryptography. Examples of secret key algorithms include the monoalphabetic cypher and the Captain Midnight code; nonetheless, breaking any of these cyphers is surprisingly simple. To use it, you need to press just one button. It also contains a message (the plaintext) in addition to the key. The encrypted data, known as ciphertext, consists of jumbled information that is about the same length as the plaintext before encryption. The same key that is employed during encryption is also used throughout the process of decryption. As seen in Figure 1 [6].

Every year, the battery industry and researchers manage to enhance the capacity of handheld devices’ batteries by only approximately 5–10%. It’s not enough juice to power mobile devices when they’re exchanging data with other mobile devices and web-based programmes. Complex processes, such as multiplication with 28 points, need a lot of power while updating the messages, files, and photographs, which is a problem for battery-powered portable devices [7].

One kind of symmetric cryptographic encryption algorithm [8] is the AES algorithm. The technique of picture segmentation [9] is one of the most difficult and important in digital image processing. Picture thresholding is a widely used method for image segmentation [10] because of its computational efficiency and low learning curve. Image thresholding works by comparing each pixel to a threshold value, which is determined automatically based on the distribution of grey levels in the picture. This allows for the separation of foreground objects from their background. Due to their usefulness in a variety of image processing and application contexts, several automated thresholding approaches have been reported in the literature [11]. Finding thresholds using metaheuristic algorithms like the BWO that are informed by the Otsu threshold technique is a viable alternative. These techniques, used at the right moment, may identify critical thresholds in a picture [8]. In this research, we suggested a method for substituting an iris biometric for a text key in the Advanced Encryption Standard (AES) algorithm’s key generation process.

- Plan the infrastructure that facilitates safe and efficient data transmission while making optimal use of available resources. In fact, this is the reason for using this research approach.
- This study demonstrates efficient Discrete Cosine Transform (DCT) with Advanced Encryption Standard (AES) algorithms [12] that might be used to transmit the data. The work is meant to assure secure data transfer without compromising efficiency.
- To make the greatest use of existing resources by suggesting strategies using the Black Widow Optimization technique that minimizes wasteful use of energy while making optimal use of what is already at hand, to maximize outcomes;

There are a few different parts to the document, and they are as follows: There is a survey and background work findings in Section 2, a suggested methodology and analysis in Section 3, results and analysis in Section 4, and a conclusion and ideas for future study in Section 5.

## 2. Literature Survey

Information security is becoming an increasingly pressing concern as wireless access networks develop, as discussed. As a result, there is a pressing need for new methods of transferring data over the internet that are more secure than the ones now in use. When it comes to wireless transmission formats, images are among the most common and commonly utilised ones. This work employs a novel approach to picture steganography, a combination of the chaos map and discrete transform techniques. Several methods of picture steganography, such as those outlined by [13] to enhance the intangibility, capability, and security of the stego image, have been developed. Due to the inherent complexity of message embedding, this analysis presents a flexible method that may choose the best model to reduce error. This flexible model, taking into account the two bits, LSB model in the compartment picture, may enhance the presentation of the opposite LSB replacement method. For some models, the error rate is calculated by testing the holder and message image bits using the opposite LSB replacement approach before the actual insertion.

A model’s low error rate determines which one is used to including the message. The LSB turnaround picture makes use of this model’s adaptability. Steganography provides a large increase in opacity. As cited [14], The central topic of this book is security, which is a necessary condition for any kind of covert communication. Even if cryptography may be used to secure data, doing so reveals the existence of hidden communication. Steganography is often used in covert mail. When doing steganography, the secret information is hidden under a cover medium to avoid suspicion. The use of a picture as a cover medium for encrypted communication is known as image Steganography. Many researchers have come up with various picture Steganography techniques. In this paper, we provide a Black Widow Optimization-based technique for high-limit image Steganography. With this novel approach, sensitive data is hidden using LSB Steganography. However, before being integrated into the cover picture LSBs, the confidential data is rearranged and modified. Adjustments and reorganization of private data are within GA’s authority. For GA to determine chromosomal value in an unconventional fashion, a novel concept known as an adaptable chromosome is introduced. When it comes to stego pictures, GA seeks to identify the optimal border esteem that will provide the highest visual quality.

Ref. [15] suggested a new method to encrypt images by using just a small number of FRFT instructions. To create an encrypted picture, many orders of inverse discrete FRFT (fractional Fourier transform) are applied to the initial image and the resulting sub-images. In addition, the insertion may be studied in the fractional Fourier domain to construct a straight framework that can be used to fully recover the original picture. If you want to encrypt images more than twice as well, you may use the method we provide.

In comparison to preexisting FRFT-based security frameworks, the suggested method has a larger key space since it makes use of the transformation orders of the FRFTs used as mystery keys. In addition, the calculation based on the fast Fourier transform can verify the encryption scheme, and the computing cost increases linearly as the key space grows. The results of the tests confirm that the picture decryption is quite sensitive to small changes in the transformation orders. The Arnold Transformation Period, as reported by [16] is a critical limit that affects the efficiency of our application in the domains of image encryption, digital watermarking, concealed data, and so on. Although the results of previous research on an alternative two-dimensional Arnold advancement are difficult to evaluate and call for practical application value, they do exist. Modelling the relationship between photo requests and the Arnold transformation’s corresponding period allowed us to collect information for a study of relapse. Table 1 shows the Comparison of Existing Methodology and their performance metrics.

Steganography requires a lot of work for relatively little quantities of hidden data. The size of the large text restricts the ability to conceal brief messages inside it. The capacity of text files is insufficient to store sophisticated data such as graphics or audio. For a steganography system to work, it requires an unauthorized observer to be able to identify. By scattering data over several sites and employing pseudonyms as IDs, the L diversity model makes it difficult for third parties to reconstruct the original data. Information loss is kept to a minimum and data usefulness is guaranteed by emphasizing suppression and splitting procedures on l diversity modelled data. The privacy study makes use of the Oldenburg and Gowalla datasets. Average location appearance ratios between 0 and 1 have been used to quantify the data’s usefulness. In retrieving information, the splitting method always returns accurate results. The suppression and splitting algorithms achieved a perfect one-hundred percent accuracy [21]. To achieve very effective data categorization while protecting sensitive characteristics, a perturbation approach based on random swapping was used. The UCI Machine Learning repository adult dataset is utilised for this study. Naive Bayes and the J48 have error rates of 0.37% and 0.32%, respectively, for 83.48% and 86.07% classification accuracy, respectively [22].

Data and privacy leaks are mitigated by using both enhanced role-based access control and cryptographic methods. As a rule, the optimized algorithm will label data as either sensitive or non-sensitive. All sensitive data was encrypted using modern technologies, and people were granted access to it according to their respective roles. Only the server, with the appropriate permissions, may decrypt the data. The user must do both the decryption and mining tasks [23] to get the original data.

Homomorphic encryption and other cryptographic methods are used to circumvent the disclosure of support and confidence. The comparison scheme between the support and confidence might have a predetermined threshold made possible by the secure comparison approach. Modular arithmetic and multiplication, as employed in the common item set mining and association rule mining, are all that is needed for this method. Performance study is conducted using the Apriori, Eclat, and FP-growth algorithms on the retail and pumsb datasets. The Apriori method takes 624 s, the Eclat algorithm takes 7 s, and the FP-growth algorithm takes 5 s to complete the same task [24].

The least lion optimization technique is used in association rule hiding to produce a secret key that is then used to convert the original dataset into a cleansed one in which the private rules are concealed. The least lion optimization strategies are used for chess, T10I4D100K, and retail datasets, yielding 84.36 percent privacy, 83.7 percent privacy, and 82.4 percent utility data, respectively. The differential privacy-based decision tree classification model uses the feedback to add a couple of noises using Laplace and exponential approaches, making the system more robust against intrusions [25].

Data security and privacy may be ensured by using these approaches to introduce noise into the system. To conduct their research, the team at UCI Machine Learning employed the Census income dataset. To determine the precision of the proposed method, the ID3, C4.5, and Diff PD algorithms are used on the dataset. ID3, C4.5, and Diff PD all have an 83% average accuracy, whereas Diff PD has an 84% average accuracy, and CPID has an 87% average accuracy. hen steganography has been used and decodes the concealed information.

Although the key must be kept secret when using symmetric encryption, the process is quick and efficient when dealing with huge volumes of data, making it preferable to asymmetric methods. However, this might be a major hassle in cases when the encryption and decryption processes are carried out in separate places and the key has to be transferred between them.

The study’s overarching goal is to aid in the creation of a powerful encryption system and a productive approach to scalar multiplication. There are two stages of Modular Multiplication in the Encryption Process. As there are more unknowns in the encryption, subsequent Known Plain Text Attacks will be challenging as well.

## 3. Proposed Methodology

User Interface, Key Distribution Centre, Initial Key Selection, Derivative Module, Key Generation Module, Encryption Module, CRT Inverse Computation Module, Cloud Database Manager, Rule Mining Module, and Decryption Module are just some of the major components that make up the overall architecture of the proposed secured data communication system. The chief among them is the cloud database administrator, who controls the whole system. 

### 3.1. Key Distribution Centre

In this work, the keys are generated by means of the key generation module, which employs a number of distinct methods and procedures. There are five main parts: prime number generation, private/public key generation, two/three-degree polynomial, GCDLCM key generation, and authentication. Each of these parts has a purpose in the larger whole. The appropriate prime numbers are generated by using the prime number creation feature. Components Private/Public Key Generation are used to produce the necessary private and public keys. In addition, the Two/Three Degree Polynomial feature will be used to implement the two-degree or three-degree polynomial value in this project. In addition, common techniques like Greatest Common Divisor (GCD) and Least Common Multiplier (LCM) must be used to construct the key (LCM). Last but not least, this section completes the authentication procedure by using the authentication component, which offers a fresh approach to authentication.

***A. Module For Encryption***

The encryption module takes data provided by the cloud user and the key creation module and encrypts it using the generated key. The Extended AES with Base 100 table, Newton’s binomial theorem, and the Improved AES with Matrix Translation are the three subcomponents that make up this module. In this study, three distinct methods of encryption are presented, and they are implemented in these subcomponents.

To encrypt the data using the key generated in conjunction with the data, the appropriate encryption method must be used.

***B. CRT Inverse Computation Module***

If you need to get the inverse value of a key, you may use this Chinese Remainder Theorem (CRT) inverse computation module. 3.8 CLOUD DATABASE MANAGER

The database is made up of IF... THEN rules.

Rules are provided for choosing one of the three security algorithms described in this work as the appropriate encryption algorithm, considering the degree of security needed by the application. The decision manager is responsible for handling rule-firing difficulties, such as exception handling and rule chaining. The decision manager triggers the rules using a forward chaining inference process, which results in smarter security and routing choices. The cluster manager, security manager, routing manager, and key distribution centre all employ the rules in the knowledge to perform their functions using deductive reasoning. By using a semantic network to represent the rules contained in the knowledge base, the system may automatically provide a taxonomy categorization of the rules kept in the database.

***C. Module for Rule Mining***

The rule mining component processes the rules in the knowledge base in response to queries from cloud users. Here, the rule mining module manages the rules according to the user’s demands, using input from the cloud database manager.

***D. Centralized Group Key Management System***

Due to fluctuations in both group membership and individual participation, the group’s central key management facility must be responsible for all key-related tasks, including the creation of new keys, their distribution, and any necessary re-keying. When the recipient is the only one with access to the encrypted material, the sender and receiver constitute a highly secure group. Multicast communication is utilised when the information is needed at intermediary nodes for purposes such as data aggregation or analysis. In a multicast setup, the key distribution centre generates and safely disseminates group keys based only on the members of the group. As a result of member join and member departure operations, it is also responsible for keeping forward and backward secrecies. It is the responsibility of a group key management agent, which is developed and deployed by the root agent, to coordinate communications between this component and the security manager and the routing manager.

***E. Decryption***

The decryption component is what actually does the decryption, using one of the many suggested decryption techniques. Each of Improved AES with Matrix Translation, nth root, and Enhanced AES with the Base 100 table constitute one of this module’s three subcomponents. The correct decryption algorithm is used in this section to reveal the encrypted data.

Digital image processing relies heavily on the ability to alter images for more effective resource management. Convolutional calculations in the Fourier Transform may be used to create images, and the Discrete Cosine Transform (DCT) can greatly decrease an image’s storage requirements without significantly impacting its visual quality. DCT [21] is used to convert an image from its spatial domain to its frequency domain. Different spectral groupings are then formed inside the picture as a result of this (as for the image’s visual quality). Locating regions of the picture with high, moderate, and low recurrence [22]. The DCT may transform a picture into a frequency domain representation after first partitioning the image’s pixel matrix into blocks of varying sizes (*N* is dependent on the kind of image). To send all of the occlusions in an 8-bit black-and-white image, for example, we set *N* = 8; for a 24-bit colour image, we use *N* = 24; however, using *N* = 24 as the block size might increase the complexity of the procedure over time. Each of the three colours (red, green, and blue) that make up a shaded image is treated separately using DCT with a block size of *N* = 8.

For a signal with *N* values in a row, the discrete cosine transform (DCT) is expressed as:

(1) $$\left(u\right)=\alpha \left(u\right)\sum_{x=0}^{N-1}\;f\left(x\right)cos\;cos\left[\frac{\pi \left(2x+1\right)u}{2N}\right]$$

For, u=0,1,2,3,………,N−1& inverse transformation is defined as

(2) $$f(x)=\sum_{x=0}^{N-1}\;C(u)\alpha (u)cos\left[\frac{\pi (2x+1)u}{2N}\right]$$

where *f*(*x*) is the signal value at point *x*&α(*u*) is the transform coefficient for value *u*.

(3) $$\alpha (u)=\{\sqrt{\frac{1}{N}},foru=0\sqrt{\frac{2}{N}},foru\neq 0$$

It is clear from (4) for u=0,

(4) $$C(u=0)=\frac{1}{N}\sum_{x=0}^{N-1}\;f(x)$$

i.e., in the above equation, the average value of the sample is represented by the last equation. The represented coefficient is represented as the DC coefficient and another coefficient, 2-D DCT, can be defined as:

(5) $$C(u,v)=\alpha (u)\alpha (v)\sum_{x=0}^{N-1}x\;\sum_{y=0}^{N-1}y\;f(x,y)\left((cos\left[\frac{\pi \left(2x+1\right)u}{2N}\right])(cos\left[\frac{\pi \left(2y+1\right)v}{2N}\right])\right)$$

for u,v=0,1,2,…,N−1. & inverse transformation is defined as

(6) $$f(x,y)=\sum_{u=0}^{N-1}x\;\sum_{v=0}^{N-1}y\;\alpha (u)\alpha (v)C(u,v)(\left(cos\left[\frac{\pi \left(2x+1\right)u}{2N}\right])(cos\left[\frac{\pi \left(2y+1\right)v}{2N}\right]\right))$$

where C(u,v) represents frequency value for u,v&f(x,y) represents pixel colour value at position (x,y).

(7) $$\alpha \left(u\right)=\sqrt{\frac{1}{N}},foru=0\;\sqrt{\frac{2}{N}},foru\neq 0$$

(8) $$\alpha (v)=\sqrt{\frac{1}{N}},forv=0\;\sqrt{\frac{2}{N}},forv\neq 0$$

### 3.2. Advanced Encryption Standard (AES) Algorithm

The National Institute of Standards and Technology (NIST) in the United States created the Advanced Encryption Standard (AES; also known as Rijndael) in (NIST). The WHO backs Vincent Rijmen and Joan Daemen, two cryptographic engineers from Belgium, for their work on the Advanced Encryption Standard (AES), a collection of Rijndael block columns.

Our group has used the tried and true AES method to persuade the government agency of the value of our concept. Since the Rijndael method is based on a set of integers, both the keys and the blocks may be any size. The government agency selected three members of the Rijndael family, each with a 128-bit block size but three different key lengths (126, 192, and 256 bits) [23].

**Figure 2:**
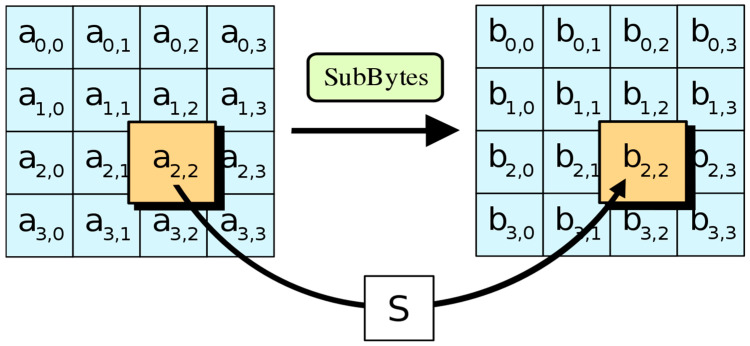
Steps of Sub Bytes.

In a bilaterally symmetrical key system, such as the one implied by the AES algorithm, the same key serves both encryption and decryption purposes. After five years of standardization work, the most successful Rijndael Figure 2 was finally shown.

The Advanced Encryption Standard (AES) is a potential global security standard that encodes and decodes 128-bit data blocks using keys of standard lengths of 128, 192, and 256 bits.

The 4 × 4 array of bytes is used to hold all of the information encrypted using AES. In this field, AES is the gold standard. For example, a 16-byte value’s bytes b0, b1,..., b15 might form a two-dimensional array:

(9) $$\left[b0\;\;\;\;b4\;\;\;\;b8\;\;\;\;b12\;b1\;\;\;\;b5\;\;\;\;b9\;\;\;\;b13\;b2\;\;\;\;b6\;\;\;\;b10\;\;\;\;b14\;b3\;\;\;\;b7\;\;\;\;b11\;\;\;\;b15\right]$$

The amount of time it takes for the input (plaintext) to be changed into the output (encoded text) in AES encryption varies with the size of the key. You’ll find several possible ways forward below.

It takes 10 iterations of the method to decode a 128-bit key.

A 192-bit key entails 12 full cycles.

Here we are at attempt number 14 with 256-bit encryption.

Beyond what is assured by the secret writing key, each tour covers a significant chunk of the process. Through a process of iterative backtracking, the text is unmasked and returned to its original form using the continuous secret writing key.

There are three possible key lengths when using cluster-based symmetric AES encoding: There is some flexibility in the bit length (up to 256), the packet size (fixed at 128), and the rules that govern the approach. Therefore, it sees widespread use across the software. Three keys totaling 128 bits in length are proposed by the AES algorithmic approach to encryption. One frequent method of measurement is the size of a key. A computational approach with 10 iterations is used to determine the length of a key. From the first round all the way to the last, there are four phases: The S-Box, the Row/Column Shift, the Main Addition, and the Final S-Box.

#### 3.2.1. The Sub Bytes Step

Each memory cell a[i, j] is swapped with a memory cell of category S, S using an 8-bit swap box in a memory substep (a[i, j]). No success can be had in achieving linear encoding using this approach. Since the square S used here is obtained by inverse multiplication in GF (28), sophisticated non-linear behaviour is to be expected on its part. Since the S-square is generated by the function in conjunction with the reverse affine conversion, it is resistant to attacks based on simple algebraic principles. Fixed inverse points and mounted points like S(ai,j) are avoided in the same way that S-Box is preferred over mounted points. To use the inverse of the byte during decryption, one must perform a backwards conversion of the probe and search for the inverse of the multiplier [17] shown in Figure 2.

#### 3.2.2. The Shift Rows Step

The Shift Rows function may be used anywhere in the globe; it moves the RAM part of the computer to the side at sporadic intervals. The largest line length in AES always remains unchanged. To refresh the whole bank, memory boards are replaced. The third and fourth lines within the same structure are compensated at two and three tiers, respectively. The symmetry technique works equally well with a 128- or 192-piece jigsaw. Because of this, the Shift Rows step uses the bytes from each segment in the information state to construct the yield state. The first line of each 256-bit block test is constant, while lines two through four may test different sets of bytes. Since AES does not make use of a key size so big, this change only affects Rijndael encryption with a block size of 256 bits.

**Figure 3:**
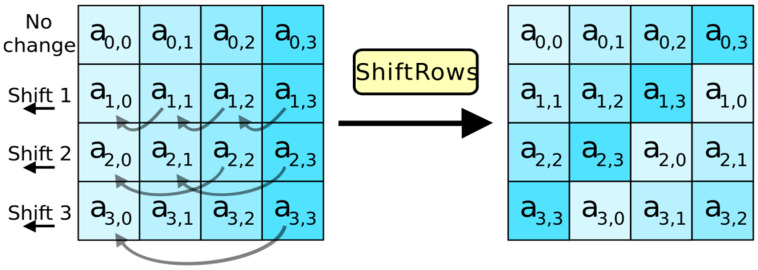
Steps of the Shift Row.

**Figure 4:**
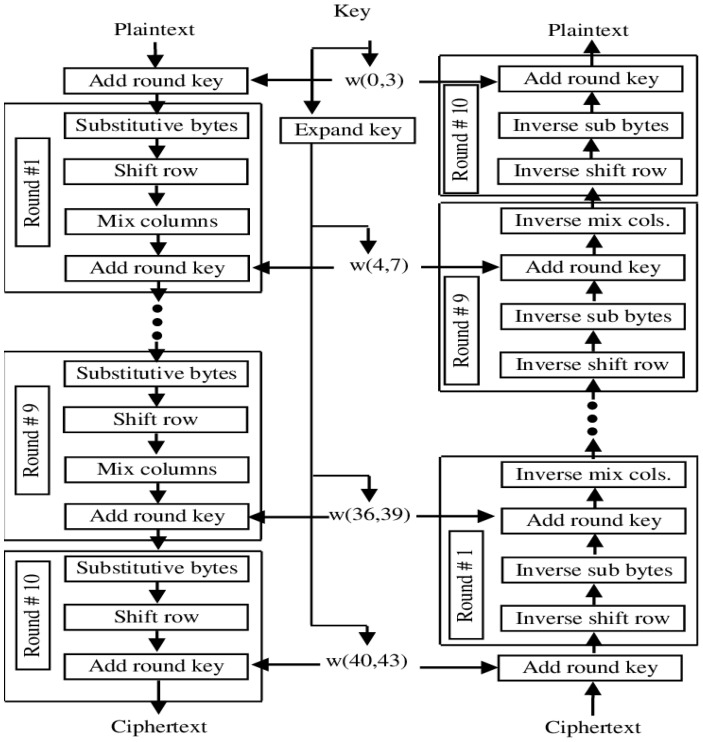
Basic AES 128-bit block diagram.

At each stage of shifting rows, bytes in each column of the state are moved to the left. The number of bytes moved in each line is different from line to line. Basic AES 128 components are shown in a block diagram in Figure 3. AES key generation is shown in Figure 4.

### 3.3. Encryption Using Galois Field Theory

For the study of irreducible polynomials over the field Q of rational numbers, it is necessary to explicitly generate a class of irreducible polynomials with the requisite Galois groups. Schur proved that if n is a positive integer, then the polynomial is a perfect square.

(10) $$f_{n}(x)=a_{n}\frac{x^{n}}{n!}+a_{n-1}\frac{x^{n-1}}{(n-1)!}+\cdots +a_{1}x+a_{0}$$

(11) $$\frac{x^{n}}{n!}+\frac{x^{n-1}}{(n-1)!}+\cdots +x+1$$

(12) $$\mu_{0i_{1}}=m_{y}^{x}\left\{\mu_{0j}|0<j⩽n|,a_{n-j}\neq 0\right\}$$

If $i_{1}<n$, let $i_{2}$ be the largest index such that $i_{1}<i_{2}⩽n$ and

(13) $$\mu_{i_{1}i_{2}}=m_{}^{}\left\{\mu_{i_{1}j}|i_{1}<j⩽n,a_{n-j}\neq 0\right\}$$

(14) $$w_{i}\left(\sum_{i}^{}\;a_{j}\theta^{j}\right)=\tilde{\tilde{v}}\left(\sum_{j}^{}\;a_{j}\theta_{i}^{j}\right),a_{j}\in K$$

Let’s call the bits of f n (x) encryption data that are spread across Q L. Let’s use the notation v _p to represent the unique extension of the p-adic valuation of the Encryption data bits Q p of p-adic numbers to the algebraic closure of Q p. Both the claim and Theorem 5.2 make this immediately obvious. In the encipherment data bit-splitting LQ p of f n (x) across Q p, the root will be found at position v _p () = 1/p. The index of ramification, and hence the degree of LQ p/Q p, which divides the degree of L/Q, are both divisible by p. Since p > n2/2, the Galois group G 0 (let’s say) of L/Q has an element of order p, which must be a p cycle, according to Cauchy’s theorem of finite groups. Since G 0 is the Galois group of an irreducible polynomial, it is also transitive. In light of this (12). For n >= 8, B that G 0 includes A n.

The slope of the rightmost edge of the Newton polygon defined by f 4 (x) = a 4 × 4/4! + a 3 × 3/3! is computed for n = 4.

+a 2 x^2/2!

In terms of the 3-adic valuation, v 3, +a 1 x + a 0. The set whose lower convex hull is the Newton polygon of f 4 (x) with regard to v3.

(15) $$S=\left\{\left(0,v_{3}\left(\frac{a_{4}}{4!}\right)\right),\left(1,v_{3}\left(\frac{a_{3}}{3!}\right)\right),\left(2,v_{3}\left(\frac{a_{2}}{2!}\right)\right),\left(3,v_{3}\left(a_{1}\right)\right),\left(4,v_{3}\left(a_{0}\right)\right)\right\}.$$

Keeping in mind that 3 does not divide a_3 and a_0 a_4 is coprime 4!, we see that

(16) $$S=\left\{\left(0,-1),(1,-1),\left(2,v_{3}\left(a_{2}\right)\right),\left(3,v_{3}\left(a_{1}\right)\right),(4,0\right)\right\}.$$

The slope of the rightmost edge of the Newton polygon of f_4 (x) with respect to v_3 according to (13) is the

(17) $$m_{\;}^{\;}\left\{-v_{3}\left(a_{1}\right),-\frac{v_{3}\left(a_{2}\right)}{2},\frac{1}{3},\frac{1}{4}\right\}=\frac{1}{3}.$$

Using the same reasoning as before, we can deduce that A 4 is contained inside G 0 since the order of G 0 may be evenly divided by both 3 and 12, as shown above.

A polynomial over K with a 0 an n0 would look like f(x) = a_n_ x_n_ + a (n_1_) x(n_1_) + a _1_ + a _0_, where K is a set of Encryption data bits with a real value v. Let’s call the plane point (i, v(a (n _i_)) *P_i_*, where (a (n_i_)) = 0, 0, n_i_). When both a (n _i_) and a (n _j_) are zero, the slope of the line connecting points *P_i_* and *P_j_* is denoted by ij. Allow i 1 to be the biggest index in the range [0, i_1_], where

(18) $$\mu_{0i_{1}}=m_{\;}^{\;}\left\{\mu_{0j}|0<j⩽n,a_{n-j}\neq 0\right\}.$$

If $i_{1}<n$, let $i_{2}$ be the largest index such that $i_{1}<i_{2}⩽n$ and

(19) $$\mu_{i_{1}i_{2}}=m_{\;}^{\;}\left\{\mu_{i_{1}j}|i_{1}<j⩽n,a_{n-j}\neq 0\right\}$$

and so on. The Newton polygon of f(x) with respect to v is the polygonal line having segments $P_{0}P_{i_{1}},P_{i_{1}}P_{i_{2}},\ldots ,P_{i_{k-1}}P_{i_{k}}$ with $i_{k}=n$.

By hypothesis, the absolute value of *d*′ defined by

(20) $$d^{\prime }=B^{n-p}+(-1{)}^{n+1}(n-p{)}^{n-p}\frac{(n!{)}^{p}}{p^{n-p}}A^{n}$$

It follows that q is a prime number and that dy may be precisely divided by an odd power. It is straightforward to verify that every prime divisor of dr (in particular p) must be coprime to n! given that *B* is coprime to n!. This fact, that p divides d K, emerges directly from Equations (19) and (20). Therefore, p is a ramified prime in K by virtue of Dedekind’s Theorem, which defines ramified primes. Then, we prove that

(21) $$g_{n}(x)\equiv (x-c{)}^{2}\phi_{2}(x)\cdots \phi_{r}(x)(modq)$$

I (x) for 2ir are monic polynomials with Z-coefficients that are both unique modulo q and irreducible modulo q. Where c is a member of Z. (a/b) will have its regular meaning in Z/qZ if and only if a/b is a rational integer in Z,q that does not divide b. If h(x) is a polynomial in Z, then h(x) will stand for the polynomial produced by replacing each coefficient of h(x) modulo q. If h(x) is not a polynomial in Z, then h(x) will stand It should be evident that to establish theorem 22 all that is required is to show that the polynomial g n (x) has precisely one multiple root and that this root, which has a multiplicity of 2, is a member of the set Z/qZ. This is the only thing that is required to do so. Every root of g _n (x) has a multiplicity that is lower than 3, which may be deduced from the fact that g _n’ (x) has no recurring components other than x, which is simple to detect. This derives from the observation that q is unable to split n!AB into smaller parts. Because D(g n (x))0, the algebraic closure of Z/qZ has a repeating root (of multiplicity 2) that we may claim corresponds to g n (x) (modq). When we first get things established

(22) $$\xi^{n-p}=-\left(n!A\right)$$

And,

(23) $$\xi^{P}=-\left(\frac{pB}{(n-p)A}\right)$$

On substituting ξ in ${g‾}_{n}^{\prime }(x)$, we have

(24) $$n‾\left(\xi^{n-1}+n!A\xi^{p-1}\right)=0.$$

Since q is coprime to n!AB, the above equation gives (24). Keeping in mind that

(25) $${g‾}_{n}(\xi )=\xi^{n}+\left(\frac{n!}{p}nA\right)\xi^{p}+n!B=0$$

On using (25), the above equation becomes

(26) $$\xi^{p}\left(-n!+\frac{n!}{p}nA\right)=-n!B$$

Cancelling n!≠0 from the above equation, we obtain (27). As p and n−p are coprime, (25) and (26) imply that ξ belongs to Z/qZ. If $\xi^{\prime }$ is another repeated root of 〖${g‾}_{n}(x)$, then by what has been shown above.

(27) $$\xi^{n-p}=\xi^{\prime n-p}=-\left(n!A\right),\xi^{p}=\xi^{\prime p}$$

And hence $\frac{\xi }{\xi^{\prime }}=1$ which proves (27).

We shall obtain the desired contradiction by showing that

(28) v(g(γ))>v(g(α)).

(29) $$v(\gamma -\alpha )=m_{\;}^{\;}\{v(\gamma -\theta ),v(\theta -\alpha )\}=v(\theta -\alpha ).$$

Let $\theta^{\left(i\right)}$ be any K-conjugate of θ. Keeping in mind (28),(29) and the fact that $v\left(\alpha -\theta^{\left(i\right)}\right)=v\left(\alpha^{\prime }-\theta \right)⩽v(\alpha -\theta )$, we have

(30) $$v\left(\gamma -\theta^{\left(b\right)}\right)⩾m_{\;}^{\;}\left\{v(\gamma -\alpha ),v\left(\alpha -\theta^{\left(i\right)}\right)\right\}=v\left(\alpha -\theta^{\left(i\right)}\right).$$

By applying (30) to the sum of all K-conjugates of, we get (31). So, the assertion may be confirmed. It follows that there is a unique _1 in K where the pair (_1 _1) is distinct.

Let f(β) be the lowest degree n1 polynomial over K. As a result, for every in K with degn 1 we have the Lemma:

(31) $$v(g(\beta ))=\frac{n}{n_{1}}v(f(\beta ))$$

It is hypothesized that there is a maximum element in the sets N j that corresponds to the value 1jn1 (g). This indicates that there is a maximal element in the set degj for 1jn 1, N j (f) = v(f())K. Based on the induction hypothesis, it can be deduced that 1jn 1 will include the most abundant element in both M j (_1) and M j (). Therefore, condition (iii) is only valid if v(1) is the biggest member of M j () for each n 1:j:n _1_ combination (ii).

Let us assume that K()/K is an extension of K that covers everything. According to Lemmn, there is a set of two numbers denoted by the symbol _1 in which the two numbers, when combined, make up a single distinct pair. By recursively using the Lemma, we can show that there exist distinct pairings of angles in the range [1 1], [2 2], and [(r _1_), r], where r is in the key of K and deg_i_ = n i. (say). In a manner that is analogous to the proof of (ii) and (iii) that was shown earlier, where n ijn (i _1_) for 1ir, we may use induction on n 0 = deg and Lemmas to demonstrate, in a matter of seconds, that max N j (g) = v(g(i)).

## 4. Experimental Results

The majority of cryptanalysis of block cyphers that work on binary sequences may be summed up as a differential attack. This is because differential attacks work by comparing two distinct sequences. Because of how common it is, the differential attack has to be taken into consideration at every stage of the creation of the cypher. If even a little change in the plain image may generate a large change in the cypher image with respect to dispersion and confusion, then the differential attack loses its efficacy and basically becomes useless. NPCR and UACI were used to determine the degree to which the encrypted image would be affected by a change made to a single pixel. In the context of picture encryption, the NPCR and UACI tests are frequently utilised to evaluate the cypher’s resistance to differential attacks. 

**Dataset:**

Standard 512 × 512 colour and grayscale pictures, including the Lenna, Baboon, Peppers, Barbara, Castle, Cameraman, and Text image, were used to evaluate the suggested approach in JAVA 1.7.0 and MATLAB 7=-.10.0 code. Using MATLAB, we enhance the same VC images with the state-of-the-art methods of today and compare their results to those of the proposed method. These state-of-the-art methods include Histogram Equalization (HE), Recursive Mean Separate HE (RMSHE), Contrast-limited adaptive HE (CLAHE), and Adjust image intensity (AII). The quality of the decrypted picture is evaluated both statistically using industry-standard metrics like AIC and CII and subjectively using human visual perception metrics like Q and the number of edges, AMBE and IEF.

The NPCR and UACI are designed to evaluate the number of changing pixels and the number of averaged changed intensities between the plaintext and ciphertext pictures, respectively, when the difference between the two sets of images is low. Even though the characteristics of these two examinations are well-defined and easy to calculate, it may be difficult to determine what a passing score really signifies in practice. Since NPCR scores may reach a maximum of 100%, it is commonly accepted wisdom that a secure cypher will have an NPCR number that is quite close to reaching this maximum. To test the known plain text assault as well as the chosen plain text assault, a cryptanalyst would attempt to change the plain picture by one bit (typically by one pixel) and then compare it to the cypher image. The attacker explores how alterations to the input may possibly result in a different image being generated by the cypher. The term “differential cryptanalysis” is used to refer to this form of analysis when discussing topics related to cryptography. Researchers use two standard criteria to measure the influence that a single pixel shift has on the final encrypted image when employing the procedures that were provided.

Number of Pixels Change Rate (NPCR)

(32) $$NPCR=\frac{\sum_{i,j}^{x}y\;\;D\left(i_{j}\right)}{W\times H}\times 100\%$$

where W and H are the width and height of ciphertext C 1 or C 2, respectively; C 1 and C 2 are two cyphertexts whose corresponding source photos differ by precisely one pixel and are the same size; where W and H are the width and height of ciphertext C 1 or C 2, respectively; C 1 (i, j) and C 2 (i, j) are notations that stand for the grayscale values of the pixels that are positioned at the grid coordinates (i, j), respectively (i, j). After that, C 1 (i, j) and C 2 (i, j) are accounted for in D(i, j) (i, j). D(i, j) = 1 if and only if C 1 (i, j) = C 2 (i, j); in any other case, D(i, j) = 0. In this particular scenario, D(i, j) is defined as

(33) $$D\left(i,j\right)=\{0,ifC_{1}\left(i,j\right)=C_{2}\left(i,j\right)\;\;\;\;\;\;1,ifC_{1}(i,j)\neq C_{2}(i,j)\}$$

The average rate of change in the brightness of similar pixels in the plain and cypher pictures is referred to as the “unified average changing intensity” (UACI) 1), and the acronym stands for “unified average changing intensity.” Encryption is made safer against differential attacks when the UACI value is increased. We make use of the following criteria to define the UACI: 2) Unified Average Frequency of Change (UACI)

(34) $$UACI=\frac{1}{W\times H}\left[\sum_{i,j}^{}\;\;\frac{C_{1}(i,i)-C_{2}(i,j)}{255}\right]\times 100\%$$

The presented algorithms have great sensitivity to changes in the fundamental picture, which is one of the attractive aspects of these algorithms. As was noted, even a seemingly little change, such as shifting the position of one pixel in the primary picture, might have a substantial effect. By making this kind of comparison between the plain picture and the cypher image, he may be able to figure out what they imply. This differential technique would be exceedingly wasteful and mostly meaningless if even a little change in the plain picture could result in a considerable change in the cypher image.

We use a 256 × 256 grayscale rendition of the photo “Lena” that can be found in the SIPI image database (http://sipi.usc.edu/db/, accessed on 1 March 2022). This serves as a baseline for our work. These are the encryption keys that are utilised: x1 0 = 0.5614, x2 0 = 0.4909, x3 0 = 0.7633, K 1 = 115.7100, K 2 = 395.0100, and K 3 = 203.4900 All of the results from the tests are obtained for a version of the basic image that is 192 pixels across and 192 pixels tall. To further assess the quality of the reconstruction, the test photo “lena” is first compressed using a variety of sampling ratios, and then the original image is reconstructed using those ratios. The peak signal-to-noise ratio, abbreviated as PSNR, is a metric that may be used to assess the overall quality of the reconstructed image. The technique for doing so is detailed below.

(35) $$PSNR=10{log}_{10}\frac{255^{2}}{1/N^{2}\sum_{i=1}^{N}\times \;\;\sum_{j=1}^{N}y\;\;[R(i,j)-X(i,j){]}^{2}},$$

In employing the nomenclature in which the reconstructed image is denoted by R(i, j) and the original picture is denoted by X(i, j). Table 2 displays the reconstructed version’s peak signal-to-noise ratio (PSNR), as well as the original ‘Lena’ image, its decrypted version at different sampling ratios, the decrypted version’s peak signal-to-noise ratio (PSNR), and 2D compressive sensing data. The PSNR of the decrypted image upon reconstruction may be found shown by the numbers in the tables.

### 4.1. Information Entropy Analysis

Using Formula (35), we find that the encrypted version of the test photo “lena” has an entropy of 7.9953 8 bits when sampled at a ratio of 56.25. This conclusion was reached after calculating the entropy.

Examining Data in a Histogram

**Figure 5:**
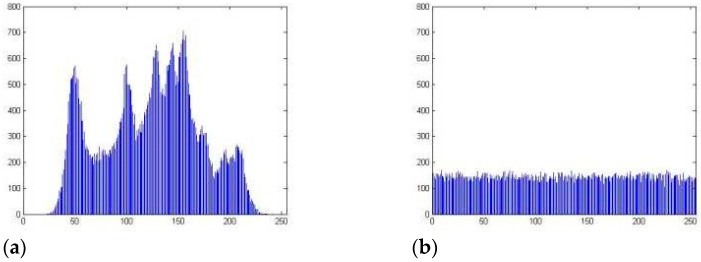
(**a**) Histogram of the plain image, (**b**) Histogram of cypher image.

An examination of the histogram may be used to determine whether or not the encryption method is resistant to being broken by utilizing statistical methods. If the encryption approach is successful, the resulting cypher picture will have an unexpected appearance and will not disclose anything about the plain image that was being encrypted. It is essential that the pixel values in the image be spread uniformly throughout the whole thing for the encryption to function correctly. The histogram of the plain picture and the cypher picture, which is shown in Figure 5a indicates that the cypher image hides all statistical information on the plain picture and Figure 5b show histogram of cipher image.

### 4.2. Correlation Analysis

In Table 3, the correlation values that were derived using a formula are shown between the plain photo “lena” and the cypher image that corresponds to it (32).

The significance of carrying out sensitivity analyses

The protection of sensitive information is the fundamental purpose of encryption, and a key is an essential component to successfully accomplish this objective. Encryption algorithms need to be constructed in such a way that the cypher image they generate is highly reliant on the key.

**Figure 6:**
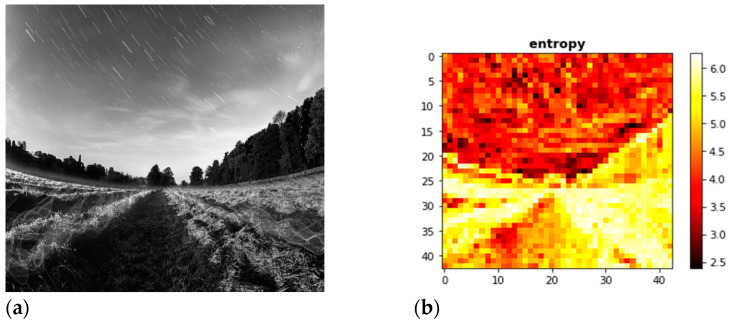
Correlation analysis: frames. (**a**) the input images, (**b**) the entropy of the image.

Figure 6a shows the input images whereas the Figure 6b shows the entropy of the image.

**Figure 7:**
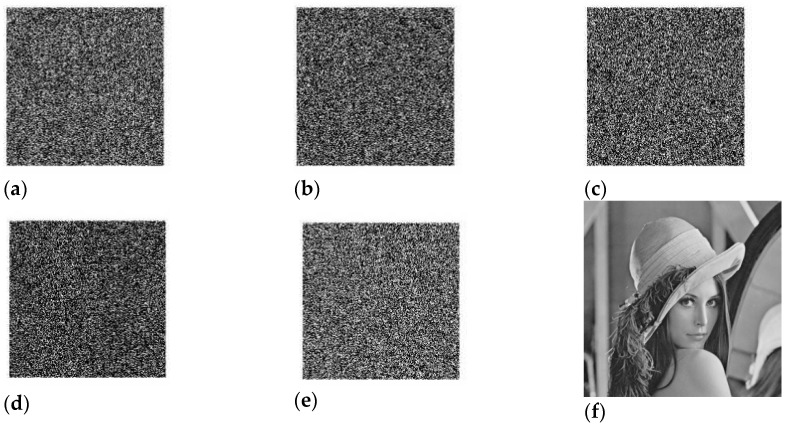
Encrypted image ‘Lena’ reconstructed using the wrong key (**a**) × 10 = 0.56 + 1.0000 × 10^−14^, (**b**) × 20 = 0.49 + 1.0000 × 10^−14^, (**c**) × 30 = 0.76 + 1.0000 × 10^−14^, (**d**) K1 = 115.7100 + 1.0000 × 10^−14^, (**e**) K2 = 395.0100 + 1.0000 × 10^−13^, (**f**) K3 = 203.4900 + 1.0000 × 10^−13^.

The “lena” photo from Table 3, which has been encrypted with a sampling ratio of 56.25, is used in the test to determine how sensitive the key is. Figure 7 depicts the image after it was rebuilt using erroneous keys that had a factor of 1014 difference between them.

**Figure 8:**
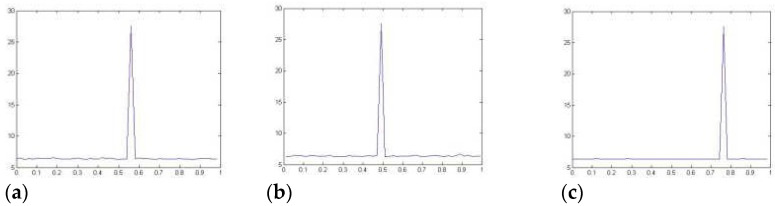
(**a**) PSNR for ×10, (**b**) PSNR for ×20, (**c**) PSNR for ×30.

A variation of the logistic map is used in the process that is being presented to produce the key sequences. It is generally agreed that the parameter and beginning point of the modified logistic map is the most important driving elements. With the help of a modified logistic map, the key sequences ×10, ×20, K1, and K2 are generated, and then these sequences are used in the calculation of the partial Hadamard matrices 1 and 2 shown in Figure 8a–c.

An array of xoring keys is generated by using the seed value ×30 and the parameter K3 in the computation. Because of this, the proposed system has an accuracy of 1014 and a total key space of around 1084, both of which are large enough to survive an attack using brute force shown in Table 4.

Here, we present a new method of secure picture sharing with an improved method of doing so that makes use of DCT-AES to enhance images. Galois field is used to reduce contrast loss in Visual Cryptography caused by pixel expansion. The findings demonstrate that SI has more integrity than traditional Visible Cryptography. Quantitative indicators such as the Contrast Improvement Index, Discrete Entropy, PSNR, Histogram analysis, Universal Image Quality Index, Edges subtracted, the Average Mean Bias of Edges, and the Inter-Edge Fuzziness Function are used to confirm the efficacy of the suggested method shown in Table 5. 

As regards PSNR and Q values, GF (CMY) often performs better than GF (RGB). Enhanced contrast is measured in terms of contrast improvement, or CII. The increased entropy of GF-enhanced images is indicative of their faithfulness to the source data. Human visual perception, a qualitative metric, also confirms the efficacy of this method. With this approach, the computational burden is lower. It works well on both colour and black-and-white photographs. Several potential fitness functions might be added to the suggested system in the future.

## 5. Conclusions

The proposed work discussed the possibility of developing an image compression-encryption method by combining Discrete Cosine Transform (DCT) with Advanced Encryption Standard (AES) with Galois field Theory. To counteract the linear nature of compressive sensing, the encryption process is nonlinear. Images in grayscale are quantized representations of the measurements that may be derived from a straightforward photograph. After going through the process of quantization, the resultant picture is then encrypted via the use of key xoring and s-box replacement. In addition to the key, the quantized values derived from the linear measurements are brought into play during the s-box exchange. Because the original, unaltered image is utilised in the process of replacing it, the encryption that results is nonlinear. This nullifies the linearity that is seen in compressive sensing. Because it is nonlinear, the approach that was offered is resistant to the plain-text attack that was stated. To analyses the effectiveness of the scheme, information entropy analysis, histogram analysis, correlation, key sensitivity analysis, and key space analysis are all used as evaluation tools. All of the research that has been presented up to this point has shown that the proposed systems are secure, but it has also shown that the various plans each have their distinct advantages. The second strategy, which makes use of dynamic s-box replacement and random diffusion, generates a greater degree of confusion and diffusion than the first one does, but it does so at the expense of a higher level of computing expense. The third method is designed to produce confusion and diffusion between the colour channels to increase the system’s resilience to differential and known/chosen-plaintext assaults. This will help the system be more secure. It can encrypt data at a rate of 0.9332 and decode data at a rate of 4.6202 per second, making it a computationally efficient method.

## 6. Discussion

It is shown that the suggested research method may achieve superior performance than current research strategies via an overall assessment carried out in a MATLAB simulation environment. The suggested technique guarantees optimum and secure data transfer by evaluating its performance primarily in terms of battery consumption level.

Computational complexity is a measure of an algorithm’s effectiveness; it is a function that provides a numerical estimate of the time and space required to apply the method. The results show that the suggested algorithms need little to no computer resources to run.

It also improves both colored and black-and-white photos equally well. When applied to optimizing problems with many variables, a fitness function that is both basic and generalizable may speed up the development of high-quality solutions. A different, more desirable result may be achieved by adjusting the input parameters and trying out other implementations of more enhanced Optimization algorithms in future work. It is possible to experiment with various fitness functions for each of these nature-inspired algorithms to see whether they may boost colour quality in certain channels. Since no mathematical operation is required to decode the secret picture before transmission, the proposed method ensures the highest level of safety, security, speed, and quality.

Even in a future workplace where small gadgets with limited resources must share information, the following measures may be taken to increase security.

More message digest algorithms can be tried out to demonstrate the performance improvement of the proposed algorithm; it is preferable to decouple the data authentication procedure from mobile devices to ensure optimal battery life; the proposed algorithm should use the minimum amount of computation possible to maximize its benefits during encrypted data transmissions.

## Figures and Tables

**Table 1:** Comparison of Existing works.

Reference	Method	Work	Performance Metrics
[14]	IDEA, AES and Blowfish	Describe the relative analysis and analysis of IDEA, AES and Blowfish for image coding and decoding.	encryption and decryption to prevent unauthorized access
[15]	RSA, KNN	described Encryption is an approach to securing undue information that not only provides guarantees, but also provides authenticity	reduces the encryption and decryption time for encrypting and decrypting the input message.
[16]	AES	Presented using the AES algorithmic rule with the most management of digital image cryptography. This methodology includes a spread of characteristics.	Better PSNR performance
[17]	GA, AES	The proposed technology uses AES and GA optimally to protect an image. It suggests a powerful technique of masking information that achieves a high level of security	achieves a high level of security, better results than previous work
[18]	GA	The co-evolutionary genetic algorithm is used to select an appropriate basis from the allowable bases of the wave packet transformation and to determine the sub bands for watermark incorporation	increase the ability to resist specific image processing methods while maintaining acceptable watermark image quality
[19]	Arnold Transform	Presented the secret exchange of digital bitmaps is studied. The digital image maps are encoded using a randomization technique with Arnold transformation, and the encrypted pictures are split to affect the secret exchange of the map	Increasing the security of the data
[20]	Modified Logistic Map	Modified Logistic Map technique for image encryption is used that shows good efficiency	Speed of faster encryption, Bigger key space

**Table 2:** NPCR and UACI Estimation.

Image	RCXA		RCSNXA		RCSXA	
	NPCR	UACI	NPCR	UACI	NPCR	UACI
Lena	99.4831%	16.6523%	99.6124%	16.688%	99.5991%	16.7293%
Girl	99.6143%	16.8576%	99.4667%	16.6589%	99.6159%	16.8135%
Baboon	99.5075%	16.7137%	99.6265%	16.7194%	99.5914%	16.8413%
Barbara	99.4663%	16.5685%	99.6124%	16.7128%	99.5968%	16.7415%
Boat	99.4492%	16.6437%	99.5972%	16.7327%	99.625%	16.7333%
Peppers	99.4812%	16.6349%	99.5995%	16.8002%	99.6151%	16.7376%
Clown	99.4938%	16.7087%	99.6025%	16.7501%	99.625%	16.7451%

**Table 3:** Correlation coefficients between adjacent pixels of plain images and cypher images.

Image	Horizontal	Vertical	Diagonal
Plain Image Lena	0.9727	0.9444	0.9188
Cipher Image Lena [proposed]	−0.0063	0.0134	−0.0126
Cipher Image Lena (Huang et al. 2014)	0.0033	0.0009	0.0058
Cipher Image Lena (Zhou et al. 2015)	0.0104	0.0299	0.0062
Cipher Image Lena (Zhou et al. 2016)	0.0042	−0.0043	0.0163

**Table 4:** Comparison of proposed work with Existing works.

Image	Enhanced Image	PSNR	Entropy (AIC)	CII	Q	No. of Edges	AMBE	IEF
Baboon	AES	6.8263	0.8510	--	0.0272	46,194	57.61	---
	ECC	5.9502	0.7498	0.2721	0.4592	60,828	112.38	0.764
	CLAHE	5.9507	0.7515	0.2722	0.4594	60,774	112.37	0.764
	AII	6.9747	1.3690	0.8371	0.8947	46,386	70.094	0.967
	RMSHE	10.7208	0.8460	0.2549	0.3504	60,928	59.56	2.360
	Proposed (RGB)	14.2234	2.642	0.33	0.3814	59,283	0.574	6.912
	PROPOSED (CMY)	14.896	3.241	0.4389	0.4	52,363	8.12	8.583
Lenna	AES	7.287	0.8615	--	0.0212	40,486	54.406	--
	ECC	6.5495	0.9581	0.3510	0.5356	54,542	105.89	0.702
	CLAHE	6.5497	0.9588	0.3510	0.5357	54,499	105.88	0.702
	AII	7.4979	1.3987	0.6348	0.7647	27,055	80.21	0.916
	RMSHE	10.3475	0.8575	0.2980	0.3726	51,169	64.43	2.025
	PROPOSED (RGB)	14.231	3.25	0.7348	0.2157	50,141	9.524	4.968
	PROPOSED (CMY)	14.459	2.6043	0.475	0.60	47,152	16.48	4.184
Peppers	AES	6.4593	0.8713	--	0.0189	32,360	69.369	--
	ECC	5.4698	1.1258	0.4727	0.6171	40,470	115.16	0.678
	CLAHE	5.4702	1.1270	0.4728	0.6173	40,365	115.15	0.678
	AII	6.4613	1.5615	0.7413	0.8537	20,379	89.745	0.863
	RMSHE	11.118	0.8690	0.2784	0.3411	31,454	46.77	2.29
	PROPOSED (RGB)	12.945	3.1741	0.6243	0.3253	31,376	21.357	4.26
	PROPOSED (CMY)	14.0113	3.049	0.568	0.47	29,802	15.992	3.677
Barbara	AES	6.2449	0.8962	--	0.0265	51,723	65.436	--
	ECC	4.8546	0.6072	0.1711	0.3808	52,853	134.36	0.668
	CLAHE	4.8547	0.6077	0.1712	0.3809	52,821	134.36	0.668
	AII	6.3658	1.3285	0.8226	0.8688	36,370	78.77	0.956
	RMSHE	11.9618	0.8962	0.2588	0.3694	51,861	45.48	3.569
	PROPOSED (RGB)	14.115	3.0245	0.3060	0.3401	41,483	14.431	6.639
	PROPOSED (CMY)	15.0658	3.0788	0.244	0.413	45,582	6.75	6.058
Castle	AES	6.6891	0.8886	-	0.0163	24,641	56.978	---
	ECC	5.1564	0.5535	0.1081	0.3559	32,890	126.83	0.68
	CLAHE	5.1565	0.5537	0.1081	0.3559	32,896	126.83	0.68
	AII	6.3409	1.1706	0.6121	0.7079	17,593	85.353	0.874
	RMSHE	11.1152	0.8886	0.20	0.3120	24,484	50.588	3.458
	PROPOSED (RGB)	12.7	3.298	0.7	0.68	25,140	16.109	4.694
	PROPOSED (CMY)	13.95	3.208	0.5205	0.18	25,691	14.813	5.762
Cameraman	AES	6.4101	0.7296	--	0.02	47,268	84.93	--
	ECC	4.6173	0.7308	0.4574	0.9801	47,271	95.608	1.042
	CLAHE	4.6177	0.7641	0.5683	0.9998	47,268	85.584	1.006
	AII	6.4101	0.7308	0.5774	1	47,268	84.771	1
	RMSHE	11.1153	0.7308	0.2667	0.4592	48,214	46.99	2.955
	PROPOSED (RGB)	11.81	2.9142	0.7148	0.67	5033	10.9	4.084
	PROPOSED (CMY)	11.92	3.0314	0.5449	0.564	48,397	10.795	3.079
TextImage	AES	12.973	0.3223	--	0. 830	36,437	13.151	--
	ECC	10.1789	0.3461	0	0.7402	0	29.670	0.526
	CLAHE	10.1789	0.3595	0	0.7402	0	29.670	0.526
	AII	12.9732	0.3461	1	1	36,437	13.151	1
	RMSHE	4.1826	0.3461	0.3098	0.5342	36,441	145.45	0.132
	PROPOSED (RGB)	13.03	3.0677	1.3579	0.2563	48,551	8.2141	1.059
	PROPOSED (CMY)	5.3089	3.098	0.8147	0.9041	38,494	93.53	1.414

**Table 5:** Comparison of Experiments Based on Accuracy.

Name	Cover Image	Secret Image	Accuracy
Experiment 1	Lena	Boat	97.24%
Experiment 2	Girl	Barbara	96.12%
Experiment 3	Baboon	Lena	94.21%
Experiment 4	Barbara	Cameraman	95.65%
Experiment 5	Boat	Girl	94.31%

## Data Availability

The datasets used during the current study are available from the corresponding author upon reasonable request.
